# State License for Nurses

**Published:** 1902-04

**Authors:** 


**A Monthly Review of Medicine and Surgery.**

*EDITOR:*

WILLIAM WARREN POTTER, M. D.

All communications, whether of a literary or business nature, books for review and exchanges should be addressed to the editor: 284 Franklin Street, Buffalo, N. Y.

Vol. XLI.—LVII. APRIL, 1902. No. 9.

### State License for Nurses.

IN KEEPING with the reforms that have been established in this country within the past few years relating to the protection of human health and life, the suggestion has been made recently that graduate nurses who propose to practise their profession shall obtain a license from the state, conferred after due examination. To this general proposition there can be no reasonable objection; but, of course, opinions will differ as to the methods of establishing it.

One of the chief difficulties to overcome is the fact that almost every hospital, public and private, in large cities and small towns, has established a training school to promote its own particular interests. In this way the public has thrust upon it all sorts of nurses with all kinds of training. Some, of course, have received adequate instruction to fit them for general nursing, and such are competent to carry out the instructions of a physician in any case whether pertaining to general or special medicine, from typhoid fever to obstetric service. These, however, have been graduated from the best training schools which, comparatively speaking, are few in number.

In the private or smaller hospitals the training is usually adapted to the requirements of the patients that are under special treatment; hence, such nurses, speaking generally, do not receive as elaborate instruction and are not as well equipped for general nursing as are those from the larger institutions. We have heard the criticism that nurses in the general and larger training schools fail to receive instruction with reference to what may be termed the niceties of nursing. The proper preparation of a tray, bringing food hot to a patient's room, the administration of a bath or the general arrangement of the patient's toilet, frequently become as important factors in the ministrations to the sick, as promptitude or adequacy in medicinal dosage. A nurse who cannot do all this and do it well has no place in the sickroom. In certain private or small hospitals, particularly those where women only are taken for treatment, greater attention is given to the refinements of nursing, and a graduate from such an institution is generally equipped in the best manner possible for private nursing among people of education.

On the other hand, the large hospitals pay considerable attention to operative procedures and other glittering generalities, but scarcely any attention to the refinements of nursing. In other words, there is no uniformity in the curriculum for the training of nurses; each hospital, public or private, establishing its own methods and standards. The result is, that this kind of instruction is in a somewhat heterogeneous state. Nurses have been admitted in many instances without adequate preliminary requirements, their training-school life has not been properly directed, and their final examinations have not been as strict as the importance of the subject demands.

It would seem to be in keeping with the spirit of the age to place the education of nurses in the hands of the regents of the University of the State of New York, and in this way make it conform to the general scheme for education, professional and otherwise, established by law. By such a plan the number of training schools could be reduced, the efficiency of others enhanced, and the quality of nurses improved. A state license is a prize that would stimulate the best efforts on the part of pupils and would lift the trade of nursing to the higher level of a profession.

